# MODILM: towards better complex diseases classification using a novel multi-omics data integration learning model

**DOI:** 10.1186/s12911-023-02173-9

**Published:** 2023-05-05

**Authors:** Yating Zhong, Yuzhong Peng, Yanmei Lin, Dingjia Chen, Hao Zhang, Wen Zheng, Yuanyuan Chen, Changliang Wu

**Affiliations:** 1 Guangxi Key Lab of Human-Machine Interaction and Intelligent Decision, Nanning Normal University, Nanning, 530001 China; 2 School of Environment and Life Science, Nanning Normal University, Nanning, 530001 China; 3 School of Computer Science, Fudan University, Shanghai, 200433 China; 4 School of Computer, Guangdong University of Petrochemical Technology, Maoming, 525000 China; 5 Department of Spleen, Stomach and Liver Diseases, Guangxi International Zhuang Medical Hospital, Nanning, 530201 China

**Keywords:** Complex disease classification, Multi-omics data integration, Graph Attention Networks, Deep learning

## Abstract

**Background:**

Accurately classifying complex diseases is crucial for diagnosis and personalized treatment. Integrating multi-omics data has been demonstrated to enhance the accuracy of analyzing and classifying complex diseases. This can be attributed to the highly correlated nature of the data with various diseases, as well as the comprehensive and complementary information it provides. However, integrating multi-omics data for complex diseases is challenged by data characteristics such as high imbalance, scale variation, heterogeneity, and noise interference. These challenges further emphasize the importance of developing effective methods for multi-omics data integration.

**Results:**

We proposed a novel multi-omics data learning model called MODILM, which integrates multiple omics data to improve the classification accuracy of complex diseases by obtaining more significant and complementary information from different single-omics data. Our approach includes four key steps: 1) constructing a similarity network for each omics data using the cosine similarity measure, 2) leveraging Graph Attention Networks to learn sample-specific and intra-association features from similarity networks for single-omics data, 3) using Multilayer Perceptron networks to map learned features to a new feature space, thereby strengthening and extracting high-level omics-specific features, and 4) fusing these high-level features using a View Correlation Discovery Network to learn cross-omics features in the label space, which results in unique class-level distinctiveness for complex diseases. To demonstrate the effectiveness of MODILM, we conducted experiments on six benchmark datasets consisting of miRNA expression, mRNA, and DNA methylation data. Our results show that MODILM outperforms state-of-the-art methods, effectively improving the accuracy of complex disease classification.

**Conclusions:**

Our MODILM provides a more competitive way to extract and integrate important and complementary information from multiple omics data, providing a very promising tool for supporting decision-making for clinical diagnosis.

**Supplementary Information:**

The online version contains supplementary material available at 10.1186/s12911-023-02173-9.

## Background

With the continuous development and refinement of high-throughput sequencing technology, a large amount of omics data has been generated, which is of great importance for people to deeply study and reveal the mystery of life. Earlier, many studies were conducted on single-omics data for disease analysis. However, due to the inherent complexity of biological systems, it is difficult to gain insight into the complex biological processes of complex diseases using single-omics data. Researchers can now easily access various levels and types of biological omics data and collect many types of biological omics data based on the same set of samples, which provides multi-omics data with unprecedented details at the molecular level for disease diagnosis and disease mechanism research [1, 2]. Compared to single-omics types, integrated analysis of multi-omics data can provide a comprehensive and in-depth study of biomedical data and can even complement any missing or unreliable information in single-omics data. It can also effectively exploit the relationships and complementary information between omics data for a broader and comprehensive analysis of complex diseases, which in turn can improve the accuracy of patient clinical outcome prediction [3–5]. Therefore, the use of multi-omics data integration techniques to analyze complex diseases has become a new direction for researchers to explore complex disease mechanisms.

Previously, researchers mainly conducted an integrated analysis of single omics based on statistical methods [6] and traditional machine learning methods [7]. However, only a fraction of the characteristics of the biological system can be captured by each omics data due to the inherent complexity of biological systems, leading to relatively one-sided results [8, 9].

In recent years, the integrated analysis of multi-omics data using new machine learning and deep learning methods has achieved state-of-the-art performance in the field of disease classification [10–12]. To improve the performance of cancer classification tasks, Ma B et al. [13] proposed an eXtreme Gradient Boosting (XGBoost) classification method to integrate the mRNA and miRNA expression data for the separation of early-stage and late-stage tumors. Lin Y et al. [14] and Elmarakeby et al. [15] proposed Deep Neural Networks Based On Multi-Omics Data (DeepMO) model and Deep Neural Network For Prostate Cancer Discovery (P-NET) model respectively to classify cancer subtypes. El-Nabawy et al. [16] proposed a Cascade Deep Forest (CDForest) to integrate multi-omics data for breast cancer subtype classification. Xu et al. [17] proposed a new Hierarchical Integration Deep Flexible Neural forest framework (HI-DFNForest) based on stacked autoencoders, which successfully subtyped invasive breast cancer, glioblastoma multiforme, and ovarian cancer using miRNA, DNA methylation, and gene expression data. However, the methods above do not consider the similarity between omics samples, resulting in limited performance improvement. To solve this problem, some researchers used graph neural networks to link omics samples in order to improve cancer classification. Wang B et al. [18] proposed a Similarity Network Fusion method (SNF) to construct network samples for each type of omics data and fuse the different graphs into a final graph, using the results for clustering, which can classify cancers into different subtypes. Ma T et al. [19] proposed an Affinity Network Fusion method (ANF) based on SNF, where ANF considers each type of omics data as a view of the patient and learns the fusion affinity matrix for clustering. Wang T et al. [20] proposed a Multi-Omics Graph Convolutional Network (MOGONET), which utilized multiple similarity graph convolutional networks to effectively integrate multi-omics data for biomedical classification. Li et al. [21] also proposed a Multi-Omics Integration Method based on Graph Convolutional Network (MOGCN) for cancer classification, whose idea is to use AE to reduce dimensionality and SNF to build a similarity network of patients, and then input feature vectors and Patient Similarity Network (PSN) into Graph Convolutional Networks (GCN) for training and testing.

In conclusion, the existing methods boosted the complex disease classification to some extent, but two issues remain unresolved. Firstly, the existing methods either rely on fully connected neural networks or utilize similarity networks. The former does not effectively exploit correlations between samples, while the latter does exploit correlations but ignores the importance of the features between samples. Secondly, Current deep learning-based methods integrate different omics data into the input or feature space, but they ignore that different types of omics data can present unique features in the high-level feature space.

To this end, we proposed a novel Multi-omics Data Integration Learning Model (MODILM) for multi-omics data integration learning to improve complex disease classification. MODILM makes full use of the latent representations learned in the exclusive subspace of each omics data. The core idea of MODILM is to use a network framework based on similarity networks, Graph Attention Networks (GAT), Multilayer Perceptron Networks (MLP), and a View Correlation Discovery Network (VCDN) to integrate and learn important feature information from multi-omics data in order to capture the specificity knowledge in single-omics data and the interrelationships of multi-omics data. So that MODILM can offer a comprehensive and rational decision for the classification of complex diseases.

The main contributions can be summarized as follows:

1. We proposed a novel feature extraction method based on cosine similarity network and GAT and MLP for omics data, which can well learn the sample-specific features and intra-association features of single-omics to produce high-level omics-specific features.
2. Based on (1) coupled with VCDN, we developed a multi-omics data integration learning model (MODILM) for improving complex disease classification. MODILM can exploit intra-omics features in the underlying subspace and the higher-level cross-omics features in the label space to provide unique class-level distinctiveness for the classification of complex diseases.
3. We conducted extensive comparison experiments against 11 baseline and state-of-the-art models on six publicly available datasets. The experimental results show that our MODILM achieves state-of-the-art performance, which demonstrates the rationality and effectiveness of MODILM.

## Materials and methods

### Method

#### Overview of MODILM

**Fig. 1:**
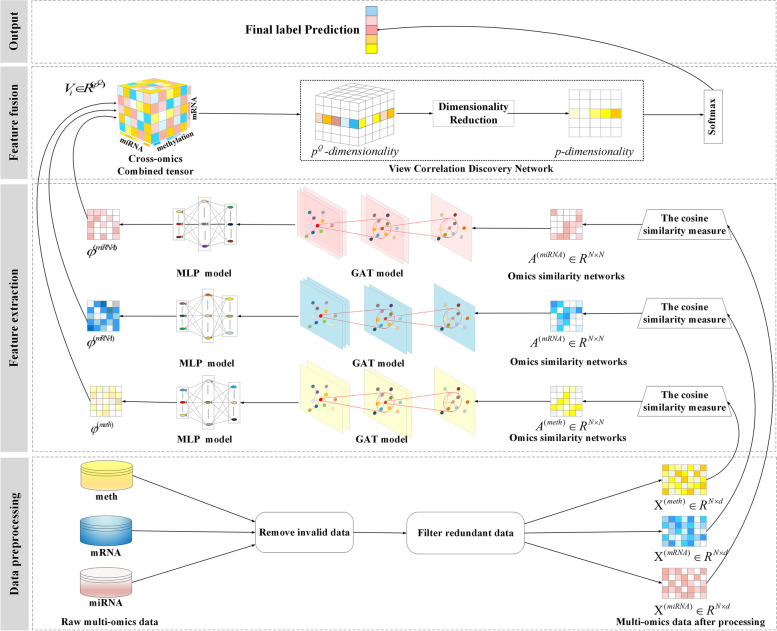
Overview of MODILM

The MODILM model is developed to better integrate and learn multi-omics features, so as to improve complex disease classification and boost biomedical diagnosis. The main working mechanism of MODILM is presented in Fig. 1. MODILM mainly consisted of three parts: a data preprocessing module, a feature extraction module, and a feature fusion module. In the data preprocessing module, we clean the original omics data to remove invalid data and redundant data. The feature extraction module includes three components: similarity network, GAT, and MLP. The similarity networks represented by adjacency matrices are constructed using cosine similarity to exploit the intra-association features of the single-omics data. GAT and MLP are used to extract features. In the feature fusion module, each omics feature representation obtained in the feature extraction module is used to construct a cross-omics discovery tensor, then a VCDN is used to fuse the features of the upper multi-omics to output the final prediction labels.

#### Data preprocessing

There are many invalid and redundant data in the original omics dataset, which can interfere with the experiment. Therefore, it is required to clean the data before conducting the experiments so as to reduce the influence of bad data on the experimental results [22]. In this section, we will introduce the specific method in the data preprocessing module.

Firstly, if the sample data belongs to one of the following two cases, it is called invalid data and needs to be removed. One case is that the sample data contains null values, NaN, INF, etc. The other case is that the data value is 0, which needs to be judged according to the proportion of 0 values in the sample data volume. If the number of 0 values is more than 10% of the total amount of sample data, the sample data will be removed.

Secondly, for redundant data, we use the ANOVA *F*-value to judge whether the sample data is redundant. Different thresholds of variance (0.1 for mRNA expression data and 0.001 for DNA methylation data) are used for different types of omics data because different types of omics data have different ranges. For miRNA expression data, we only remove samples with the ANOVA *F*-value of 0 due to its relatively small amount of data and limited available samples. In addition, we use the ANOVA *F*-value to assess the relationship between different samples in the same dataset. The specific processing procedure is as follows. For each classification task, we calculate the ANOVA *F*-value of its sample features to evaluate whether the features are significantly different between various categories. The size of the ANOVA *F*-value determines the number of features obtained after data filtering. However, if the number of features is too large, it will introduce too much noise into the model. If it is too small, the model will be unable to learn complementary information about features. Therefore, we finally decide to use data with ANOVA *F*-values smaller than 0.5.

After the above two steps of data processing, one can get the data that better express the omics information.

#### Feature extraction

In order to effectively obtain more representative features from the omics data and improve the performance of the model prediction, we designed a feature extraction module in this work. To extract the internal relationships and features of single-omics data, this work treats each data sample of the single-omics data as a node in a similarity network of the omics data. We first introduce cosine similarity in MODILM and set a threshold to construct a similarity network for each omics, so as to preliminarily judge the degree of correlation of node features and obtain topological information of omics data. Then, we use GAT to extract structural information and important features of the omics, and finally feed the obtained features into MLP to uniformly map them into a new feature space. In this way, it can further enhance and extract the high-level omics-specific features of omics data and produce better single-omics representation vectors.

1. Construction of omics Similarity Networks

To preliminarily assess the degree of correlation between node features and capture the topological information in omics data, MODILM first calculates the similarity between node pairs using the cosine similarity measure. It constructs a similarity network using the original adjacency matrix ${\mathbf{A}}$ and then retains the edges with cosine similarity greater than a given threshold $\theta$. Here, $A_{ij}$ is the adjacency relationship between node $i$ and node $j$, and the calculated results are shown in Eqs. (1) and (2).

1 $$A_{ij} = \left\{ {\begin{array}{*{20}c} {{\text{C}} \left( {x_{i} ,x_{j} } \right),} & { \cdot {\text{if }}i \ne j{\text{ and }}{\text{C}} \left( {x_{i} ,x_{j} } \right), \ge \theta } \\ {0,} & {\text{ otherwise }} \\ \end{array} } \right.$$

2 $${\text{C}} \left( {x_{i} ,x_{j} } \right) = \frac{{x_{i} \cdot x_{j} }}{{\left\| {x_{i} } \right\|_{2} \left\| {x_{j} } \right\|_{2} }}$$

where, $x_{i}$ and $x_{j}$ are the feature vectors of node $i$ and node $j$, and ${\text{C}} \left( {x_{i} ,x_{j} } \right)$ denotes the cosine similarity between node $i$ and node $j$. The threshold $\theta$ is determined by the parameter $k$ which denotes the average number of edges retained by each node, including self-connections. $k$ is calculated as Eq. (3).

3 $$k = \frac{{\sum\limits_{i,j} f \left( {{\text{C}} \left( {x_{i} ,x_{j} } \right) \cdot \ge \cdot \theta } \right)}}{N},k \in \left\{ {2 \le k \le 10,k \in N^{ + } } \right\}$$

where, $f( \cdot )$ is an indicator function and $N$ is the number of nodes. Note that the same value of $k$ is used for all experiments on the same dataset.

(2) GAT + MLP extracts rich omics features

A key issue in the classification of complicated diseases is how to obtain the features of nodes and the relationships between nodes in the omics data. GAT is able to solve this problem well, as it can naturally integrate the node features and topological information in the whole network and capture them without needing to know the structure of the network in advance [23]. Moreover, GAT implements adaptive matching of different neighboring node weights based on the trained multi-head self-attention mechanism, which makes adaptive aggregation of neighboring features possible [24]. There are dynamic changes in human cells throughout the life process, and the adaptive weight matching of GAT can better simulate the dynamic refinement of omics data interactions [25]. Therefore, we use GAT to learn rich features of nodes and topology from single-omics data, which describe sample-specific features and correlation features of the single-omics data.

In this work, GAT learns high-level features of nodes in a graph mainly by applying a multi-headed self-attention mechanism, where each attention head has its own parameters. Assuming there are $N$ nodes in the graph, the output of GAT can be expressed as Eq. (4).

4 $$H^{\prime } = \left[ {h_{1}^{\prime } , \ldots ,h_{i}^{\prime } , \ldots ,h_{N}^{\prime } } \right]$$

The output feature of each node $h_{i}^{\prime }$ is shown in Eq. (5).

5 $$h_{i}^{\prime } = \mathop {\text{Concat}}\limits_{m = 1, \ldots ,M} \left( {\alpha_{i,i}^{m} {\mathbf{W}}h_{i} + \sum\limits_{j \in N(i)} {\alpha_{i,j}^{m} } {\mathbf{W}}h_{j} } \right)$$

where, ${\text{Concat}} ( \cdot )$ denotes the concatenation function, $m$ is the number of attention heads, ${\mathbf{W}} \in {\mathbb{R}}^{{F^{^{\prime}} \times F}}$ is a weight matrix, $F$ denotes the number of input features per node, and $F^{^{\prime}}$ denotes the number of output features per node. The attention factor $\alpha_{i,j}$ between each input node $i$ and its first-order nearest neighbor $j$ in the graph is calculated as shown in Eq. (6).

6 $$\alpha_{i,j} = \frac{{\exp \left( {{\text{ LeakyRelu }}\left( {a^{T} \left[ {{\text{Concat}} \left( {{\mathbf{W}}h_{i} ,{\mathbf{W}}h_{j} } \right)} \right]} \right)} \right)}}{{\sum\limits_{k \in N(i)} {\exp } \left( {{\text{LeakyRelu}} \left( {a^{T} \left[ {{\text{Concat}} \left( {{\mathbf{W}}h_{i} ,{\mathbf{W}}h_{k} } \right)} \right]} \right)} \right)}}$$

where, $h_{i}$ denotes the input features of the node $i$, as shown in Eq. (7).

7 $$h_{i} = \sigma \left( {{\tilde{\mathbf{A}}}h_{i - 1} {\mathbf{W}}_{i - 1} } \right)$$

8 $$\tilde{\mathbf{A}} = \hat{\mathbf{D}}^{\boldsymbol{-}\frac{\mathbf{1}}{\mathbf{2}}} \hat{\mathbf{A}}\hat{\mathbf{D}}^{\boldsymbol{-}\frac{\mathbf{1}}{\mathbf{2}}} \boldsymbol{=}\hat{\mathbf{D}}^{\boldsymbol{-}\frac{\boldsymbol{1}}{\boldsymbol{2}}} {\mathbf{(A + I)}\hat{\mathbf{D}}}^{{\boldsymbol{-}}\frac{{\mathbf{1}}}{{\mathbf{2}}}}$$

where, ${\hat{\mathbf{D}}}$ is the ${\hat{\mathbf{A}}}$ diagonal nodal degree matrix, ${\mathbf{I}}$ is the unit matrix, and ${\mathbf{A}}$ is calculated from Eq. (1). We constructed one multi-layer GAT for each type of omics data. For the $q{\text{ - th}}$ type of omics data, the corresponding GAT is trained using the training data ${\mathbf{X}}^{(q)} \in {\mathbb{R}}^{N \times d}$ and the corresponding adjacency matrix ${\tilde{\mathbf{A}}}^{(q)} \in {\mathbb{R}}^{N \times N}$ calculated from Eq. (8). The output feature ${\mathbf{Y}}^{(q)}$ containing the feature information of the node and topology is shown as Eq. (9).

9 $${\mathbf{Y}}^{(q)} = GAT\left( {{\mathbf{X}}^{(q)} ,{\tilde{\mathbf{A}}}^{(q)} } \right)$$

To build more expressive and discriminating representations of omics data, MLP is introduced to uniformly map the node and topological feature vectors obtained from GAT into a new feature space using for further integration processing, as shown in Eq. (10).

10 $${\mathbf{\varphi }}^{(q)} = MLP\left( {{\mathbf{Y}}^{(q)} } \right)$$

where, ${\mathbf{\varphi }}^{(q)}$ is the final feature for each type of omics data. Eq. (10) can be formalized in detail as Eq. (11).

11 $${\hat{\mathbf{Y}}}_{{{\mathbf{t + 1}}}} = \left\{ {\begin{array}{*{20}c} {\sigma \left( {{\hat{\mathbf{Y}}}_{{\mathbf{t}}} {\mathbf{w}}_{{\mathbf{t}}} + b_{t} } \right),t > 0} \\ {{\mathbf{Y}}^{{{\mathbf{(q)}}}} {\mathbf{w}}_{{\mathbf{t}}} ,t = 0} \\ \end{array} } \right.$$

where, $t$ is the number of hidden layers in MLP, ${\hat{\mathbf{Y}}}_{{\mathbf{t}}}$ is the input data $t$-the layer, ${\mathbf{w}}_{{\mathbf{t}}}$ is the trainable weight matrix,  $b_{t}$ is the bias, $\sigma$ is an activation function (LeakyReLU function was used in our experiments).

In this work, the cross-entropy loss function was used to calculate the loss of the GAT + MLP phase. Specifically, considering the label imbalance in the training data, we further applied different weights to different categories of losses, and the weights of each category respectively correspond to their frequency in the training data. Therefore, the final loss is shown in Eq. (12).

12 $$L_{GAT + MLP} = - \sum\limits_{q}^{Q} {\left( {\frac{{{\mathbf{S}}\left( {\sum\limits_{i}^{N} {{\mathbf{y}}_{i}^{(q)} } \log \left( {{\mathbf{\varphi }}_{i}^{(q)} } \right)} \right)}}{N}} \right)}$$

where, $Q$ denotes the number of omics types, $N$ denotes the total number of samples, ${\mathbf{y}}_{i}^{(q)}$ denotes the probability distribution of the true label for the $i{\text{ - th}}$ sample of the $q{\text{ - th}}$ type of omics data, and ${\mathbf{\varphi }}_{i}^{(q)}$ is the probability distribution of the predicted label for the $i{\text{ - th}}$ sample of the $q{\text{ - th}}$ type of omics data. ${\mathbf{S}} \in {\mathbb{R}}^{1 \times N}$ is a matrix that describes the frequency of occurrence of different categories. It is calculated as Eq. (13).

13 $$S_{\rho } = \frac{{{\text{count}} (\rho )}}{N},\rho \in classes$$

where, $\rho$ denotes the number of the corresponding category of the label, ${\text{count}} (\rho )$ denotes the total number of occurrences of class $\rho$ in the label. $classes$ denotes the total number of categories.

#### Feature fusion

Different types of omics data can be considered as different views of the patient. Therefore, the fusion of multiple omics data can obtain correlated or complementary information from different views of the patient. Multi-view data fusion can eliminate redundant information arising from the correlation between different feature sets and help improve the model performance [26]. However, the existing methods for fusing multi-view data mainly involve concatenating features from different views [27], integrating weighted features from each view [28], and fusing different features in low-dimensional space [29]. They ignore the correlation between different multi-view data. In this work, we transferred the VCDN approach [30] from the image research field to the omics data research field to fuse different omics data and perform classification, considering the correlation between different multi-view data.

VCDN is used to learn the relevance of higher-level intra-view and cross-views in the label space. Although the original VCDN was designed for samples with two views, we generalize it to $Q$ views in this work. For each sample of $Q$ types of omics data, we construct a cross-omics discovery tensor $V_{i} \in {\mathbb{R}}^{{p^{Q} }}$ for $i$-th sample, where each term of $V_{i}$ is calculated as Eq. (14).

14 $$V_{{i,a_{1} a_{2} ...a_{Q} }} = \prod\limits_{q = 1}^{Q} {\varphi_{{i,a_{q} }}^{(q)} } ,q = 1,2,...,Q$$

where, $\varphi_{i,a}^{(q)}$ denotes the $a{\text{ - th}}$ term of ${\mathbf{\varphi }}_{i}^{(q)}$ omics data. We reshape the cross-omics discovery tensor $V_{i}$ to a $p^{Q}$ dimensional vector $\beta_{i}$, then feed it into $VCDN( \cdot )$ to produce a logits vector $z_{i}$, which is formulated as Eq. (15). The loss function of $VCDN( \cdot )$ is shown in Eq. (16), which is used for training $VCDN( \cdot )$.

15 $$z_{i} = VCDN(\beta_{i} )$$

16 $$L_{VCDN} = \sum\limits_{i = 1}^{N} {L_{CE} } ({\text{softmax}} (z_{i} ),y_{i} )$$

where, $L_{CE} ( \cdot )$ is the cross-entropy loss function and $y_{i}$ denotes the true labels of multi-omics data.

In this work, we use the miRNA expression, mRNA, and DNA methylation data $(Q = 3)$ to experiment, and we construct a cross-omics discovery tensor $V_{i} \in {\mathbb{R}}^{p \times p \times p}$, where each term of $V_{i}$ is calculated as shown in Eq. (17). Thus, $VCDN( \cdot )$ can integrate three types of omics data and learn potential cross-view label correlations, helping performance improvements of complex disease classification.

17 $$V_{{i,a_{1} a_{2} a_{3} }} = \varphi_{{i,a_{1} }}^{(1)} \varphi_{{i,a_{2} }}^{(2)} \varphi_{{i,a_{3} }}^{(3)}$$

Finally, the total loss function for MODILM is formulated as Eq. (18).

18 $$L = \min (\sum\nolimits_{q = 1}^{3} {L_{GAT + MLP}^{(q)} } + \gamma L_{VCDN} )$$

where, $\gamma$ is the trade-off parameter between the omics feature extraction loss and the final omics loss of $VCDN( \cdot )$. During training, we train each omics data through GAT + MLP, so as to fit $VCDN( \cdot )$ more closely and minimize the loss function $L$. To this end, MODILM can learn both the higher-level intra-view and cross-view correlations in the label space, providing unique class-level distinctiveness.

### Benchmark datasets

In this paper, we use six publicly available biomedical datasets to demonstrate the effectiveness and advantages of the proposed model. These six benchmark datasets include the Alzheimer's disease dataset (ROSMAP), the LowGrade Glioma binary classification dataset (LGG-2), the LowGrade Glioma multi-classification dataset (LGG-4), the Breast Cancer dataset (BRCA), the Melanoma dataset (SKCM), and the Lung Squamous Cell Carcinoma datasets (LUSC). The different omics data in the ROSMAP dataset are got from the AMP-AD Knowledge Portal [31]. Different omics data in LGG, BRCA, SKCM, and LUSC are got from the TCGA public data at http://xena.ucsc.edu/, which is produced by the Johns Hopkins University and the University of Southern California TCGA Cancer Genome Representation Center [32, 33].

Specifically, the ROSMAP datasets, which are made up of ROS and MAP and both from the Rush University Longitudinal Clinicopathology Cohort Study of AD, are utilized to classify patients with Alzheimer's disease (AD) versus normal controls (NC) [34]. LGG datasets are used to grade low-grade gliomas, as generally four grades: grade I, II, III, and IV, according to the World Health Organization (WHO) classification [35, 36]. In actual clinical practice, they can be divided into two categories, low order (Low) and high order (Hight), depending on their malignancy [37]. Therefore, in this paper, we divide LGG datasets into these two cases for discussion. BRCA datasets are used to classify the PAM50 subtypes of breast cancer, consisting of five categories: Normal-like, Basal-like, HER2-enriched, Luminal A, and Luminal B [38, 39]. SKCM datasets are used for the classification of melanoma, consisting of the Keratin, Immune, and MITF-low categories [40, 41]. LUSC datasets are used for the classification of lung squamous cell carcinoma, including the categories Basal, Classical, Secretory, and Primitive [42].

In this study, we explore the use of three types of omics data for classification, including miRNA expression, mRNA, and DNA methylation data for matched samples. Details of the dataset are listed in Table 1. Since noisy redundant features might degrade classification performance, we must pre-process each omics dataset independently. Since noisy redundant features may affect the performance of the classification task, we need to perform preprocessing for each type of omics data separately. The resulting number of features used for training is also listed in Table 1. The "Number of features for training" refers to the number of features comprising different omics types in the same sample.

**Table 1:** Description of the dataset

Dataset	Categories	Number of features			Number of features for training		
		**miRNA**	**mRNA**	**meth**	**miRNA**	**mRNA**	**meth**
ROSMAP	NC:169, AD:182	309	55889	23788	200	200	200
LGG-2	Low:210, Hight:311	2158	20531	485578	557	2000	2000
BRCA	Normal-like:115, Basal-like:131, HER2-enriched:46, Luminal A:436, Luminal B:147	2239	20531	485578	503	1000	1000
SKCM	Keratin:98, Immune:163, MITF-low:59	2221	20531	485578	235	2000	2000
LGG-4	I:146, II:138, III:324, IV:120	2158	20531	485578	557	2000	2000
LUSC	Basal:10, Classical:16, Secretory:18, Primitive:8	2214	20531	485578	296	2000	2000

The second column is the type of samples contained in the dataset. Also, miRNA in the table refers to miRNA expression data. mRNA refers to mRNA expression data. meth refers to DNA methylation data

### Experimental setup

#### Evaluation method

We adopted the evaluation metrics used in previous studies[43, 44], including accuracy (ACC), F1 score (F1), and area under the receiver operating characteristic curve (AUC), to evaluate the performance of the proposed model against 11 comparative models. To make a fair comparison, we used ACC, average F1 score weighted support (F1-weighted), and macro F1 average score (F1-macro) for the multiclass classification task.

#### Comparative models

We compared the performance of MODILM with three baseline models and eight state-of-the-art models. The baseline models include K-Nearest Neighbor (KNN), Support Vector Machine (SVM), and Random Forest (RF). The state-of-the-art models include Block PLSDA [45], a fully connected Neural Network classifier (NN) [46], XGBoost [13], DeepMO [14], CDForest [16], P-NET [15], MOMA [47], and MOGONET [20]. Among these, DeepMO, CDForest, P-NET, MOMA and MOGONET are deep learning models. The following is a brief introduction of the state-of-the-art models follows.

(I) Block PLSDA. Block PLSDA is a multi-omics integration method that projects data to latent structures with discriminant analysis, which integrates multiple omics data measured on the same set of samples to classify a discrete outcome.
(II) NN. NN is a fully-connected neural network classification model based on the principle of feature learning using multiple fully-connected layers.
(III) XGBoost. XGBoost is an extreme gradient boosting tree for classification that continually generates new trees which are learned based on the difference between the previous tree and the target value.
(IV) DeepMO. DeepMO is a deep neural network that learns features after integrating multiple datasets, and finally classifies the results.
(V) CDForest. CDForest model uses a multi-level cascaded deep forest structure to learn features and then concatenates the results of each forest for classification.
(VI) P-NET. P-NET is a feed-forward neural network model with nodes and edges constraints that learns custom paths, gene sets and modules, and then makes classification predictions.
(VII) MOMA. MOMA consists mainly of a module encoder, module attention mechanism, and fully connected layer. Features are learned in three stages and finally classified for prediction.
(VIII) MOGONET. MOGONET is composed of GCN and cross omics tensor discovery module, which learns the features of different omics through the GCN, then integrates the data through the Cross Omics Tensor Discovery module and finally makes classification predictions.

### Experimental settings

In our experiment, the feature extraction module consists of 2 GAT layers and an MLP with 2 hidden layers. Among them, the number of multi-head attention heads in each GAT layer is 8, and the LeakyReLU activation function is used after each GAT layer. The learning rate is set to 0.0001, and $\gamma$ is set to 1. Adam is used as the optimization algorithm to train the network. For ROSMAP, LGG-2, BRCA, SKCM, LGG-4, and LUSC datasets, $k$ (the average number of edges retained by each node) is set to 5, 8, 10, 2, 2, and 3, respectively.

### Experimental results

In this section, we first compare our MODILM against other existing models on the six benchmark datasets, then investigate the performance of MODILM under different types of omics data. Next, to find their optimal values, we evaluate the performance impact of some key hyperparameters in the experiment, such as the average number of edges retained by each node, the number of GAT layers, and the number of hidden layers in the MLP. Finally, we conduct an ablation study to investigate the impact of GAT and VCDN on MODILM’s performance.

### Comparison of experimental results with existing methods on different datasets

1. Results on the binary classification task dataset

Table 2 shows a comparison between MODILM and other existing methods on ROSMAP and LGG-2 datasets. The results show that our MODILM achieves the best performance on both ROSMAP and LGG-2 datasets. Among the existing methods, MOMA and MOGONET obtain the best performance on the ROSMAP and LGG-2 datasets, respectively, while KNN has the worst classification performance on both of these two datasets. Our MODILM outperforms MOMA by 2.5%, 2.4%, and 1.6% in terms of ACC, F1, and AUC on the ROSMAP dataset and surpasses MOGONET by 2.4%, 2%, and 3.2% in terms of ACC, F1, and AUC on the LGG-2 dataset. On the other hand, our MODILM achieves 18.6%, 17.9%, and 18.2% higher performance than KNN in terms of ACC, F1, and AUC on the ROSMAP dataset, and achieves 24.6%, 24%, and 19.4% better performance than KNN in terms of ACC, F1, and AUC on the LGG-2 dataset, respectively.

**Table 2:** Comparison results on the datasets of binary classification tasks

**Method**	ROSMAP dataset			LGG-2 dataset		
	**ACC**	**F1**	**AUC**	**ACC**	**F1**	**AUC**
KNN	0.657	0.671	0.709	0.729	0.738	0.799
SVM	0.770	0.778	0.770	0.737	0.748	0.810
RF	0.726	0.734	0.811	0.756	0.767	0.840
block PLSDA	0.742	0.755	0.830	0.729	0.738	0.799
NN	0.755	0.764	0.826	0.754	0.757	0.754
XGBoost	0.760	0.791	0.837	0.748	0.742	0.823
DeepMO	0.772	0.780	0.801	0.765	0.760	0.786
CDForest	0.778	0.791	0.839	0.843	0.858	0.871
P-NET	0.805	0.810	0.818	0.886	0.890	0.897
MOMA	0.818	0.826	0.875	0.942	0.939	0.950
MOGONET	0.815	0.821	0.874	0.951	0.958	0.961
**Our MODILM**	**0.843**	**0.850**	**0.891**	**0.975**	**0.978**	**0.993**

In conclusion, our MODILM wins the best on the datasets of binary classification tasks and achieves a lot of improvements compared to state-of-the-art methods.

(2) Results on the multi-classification tasks

The comparison results between MODILM and other existing methods on four datasets of multi-classification tasks are shown in Table 3. The results show that our MODILM outperforms all comparative methods across all the metrics on all datasets.

**Table 3:** Comparison results on the datasets of multi-classification tasks

**Method**	**BRCA dataset**			**SKCM dataset**			**LGG-4 dataset**			**LUSC dataset**		
	**ACC**	**F1-weighted**	**F1-macro**	**ACC**	**F1-weighted**	**F1-macro**	**ACC**	**F1-weighted**	**F1-macro**	**ACC**	**F1-weighted**	**F1-macro**
KNN	0.742	0.729	0.682	0.772	0.767	0.736	0.739	0.738	0.741	0.722	0.728	0.689
SVM	0.729	0.702	0.640	0.813	0.812	0.805	0.751	0.750	0.754	0.735	0.732	0.598
RF	0.755	0.733	0.649	0.859	0.857	0.827	0.756	0.742	0.733	0.722	0.838	0.524
Block PLSDA	0.642	0.534	0.369	0.860	0.861	0.830	0.76	0.758	0.772	0.754	0.748	0.751
NN	0.754	0.740	0.668	0.847	0.856	0.862	0.789	0.788	0.786	0.766	0.778	0.781
XGBoost	0.781	0.764	0.701	0.881	0.880	0.863	0.810	0.809	0.798	0.778	0.825	0.741
DeepMO	0.782	0.750	0.723	0.855	0.835	0.837	0.821	0.826	0.835	0.771	0.776	0.780
CDForest	0.789	0.756	0.759	0.862	0.851	0.842	0.878	0.886	0.891	0.778	0.781	0.783
P-NET	0.785	0.776	0.712	0.875	0.861	0.865	0.889	0.897	0.901	0.780	0.791	0.782
MOMA	0.816	0.811	0.790	0.905	0.891	0.886	0.939	0.932	0.926	0.839	0.835	0.810
MOGONET	0.829	0.825	0.774	0.913	0.913	0.912	0.943	0.942	0.927	0.855	0.838	0.799
**Our MODILM**	**0.845**	**0.840**	**0.804**	**0.928**	**0.927**	**0.925**	**0.954**	**0.954**	**0.948**	**0.865**	**0.855**	**0.833**

Among the existing methods, MOGONET achieves the best performance on all datasets except for MOMA, which achieves the best F1-macro on the BRCA and LUSC datasets. Compared to state-of-art models, our MODILM obtains 1.0% to 21.5%, 1.2% to 21.6%, and 1.3% to 43.5% improvement in terms of ACC, F1-weighted, and F1-macro on the multi-classification tasks, respectively.

In a word, our MODILM outperforms state-of-the-art methods on all the datasets of multi-classification tasks.

### Performance of MODILM with different types of omics data

**Fig. 2:**
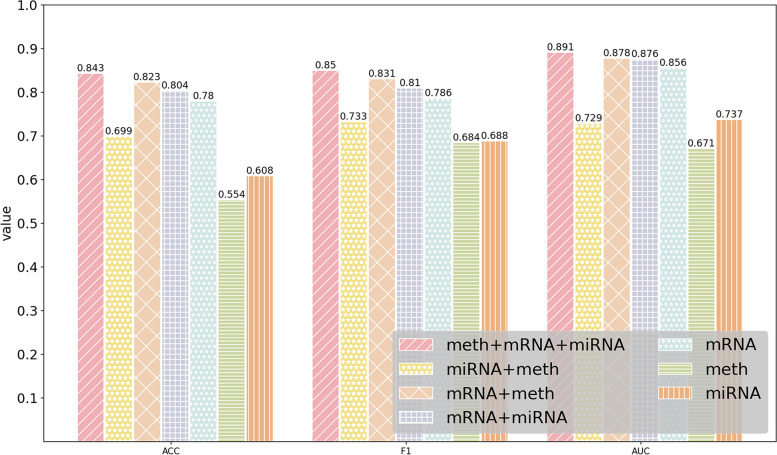
Performance of MODILM with different types of omics data in the ROSMAP dataset

**Fig. 3:**
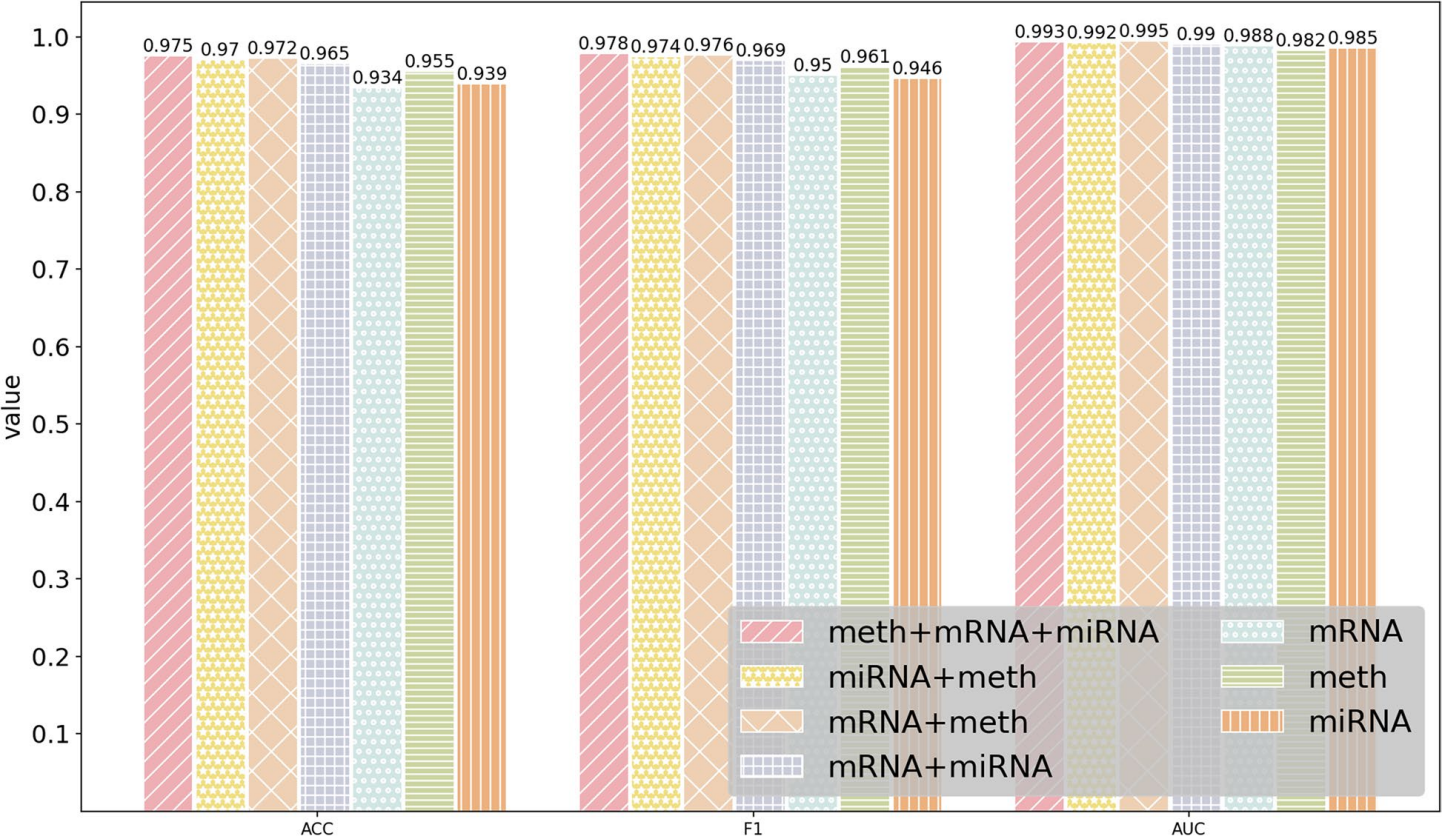
Performance of MODILM with different omics data types in the LGG-2 dataset

**Fig. 4:**
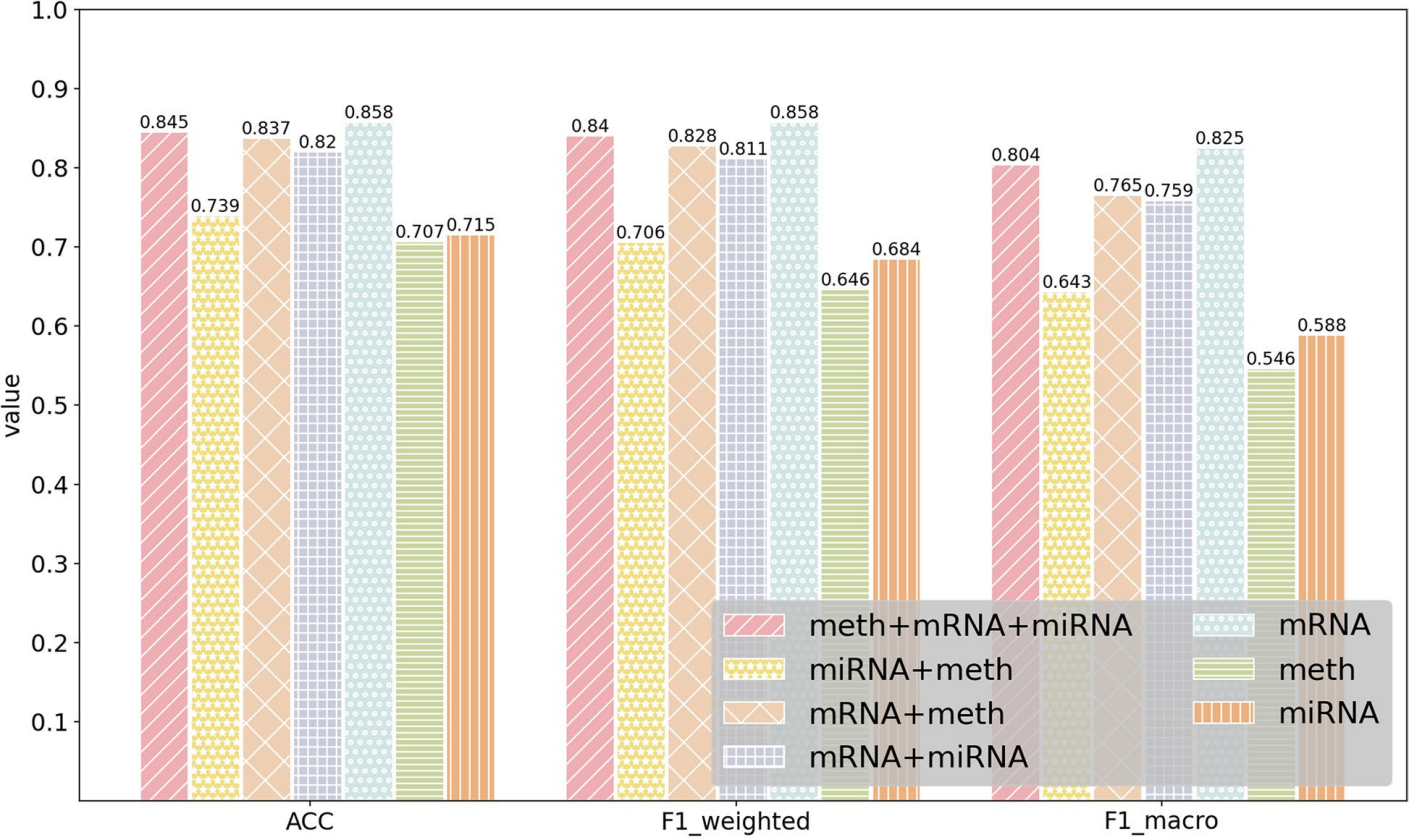
Performance of MODILM with different omics data types in the BRCA dataset

**Fig. 5:**
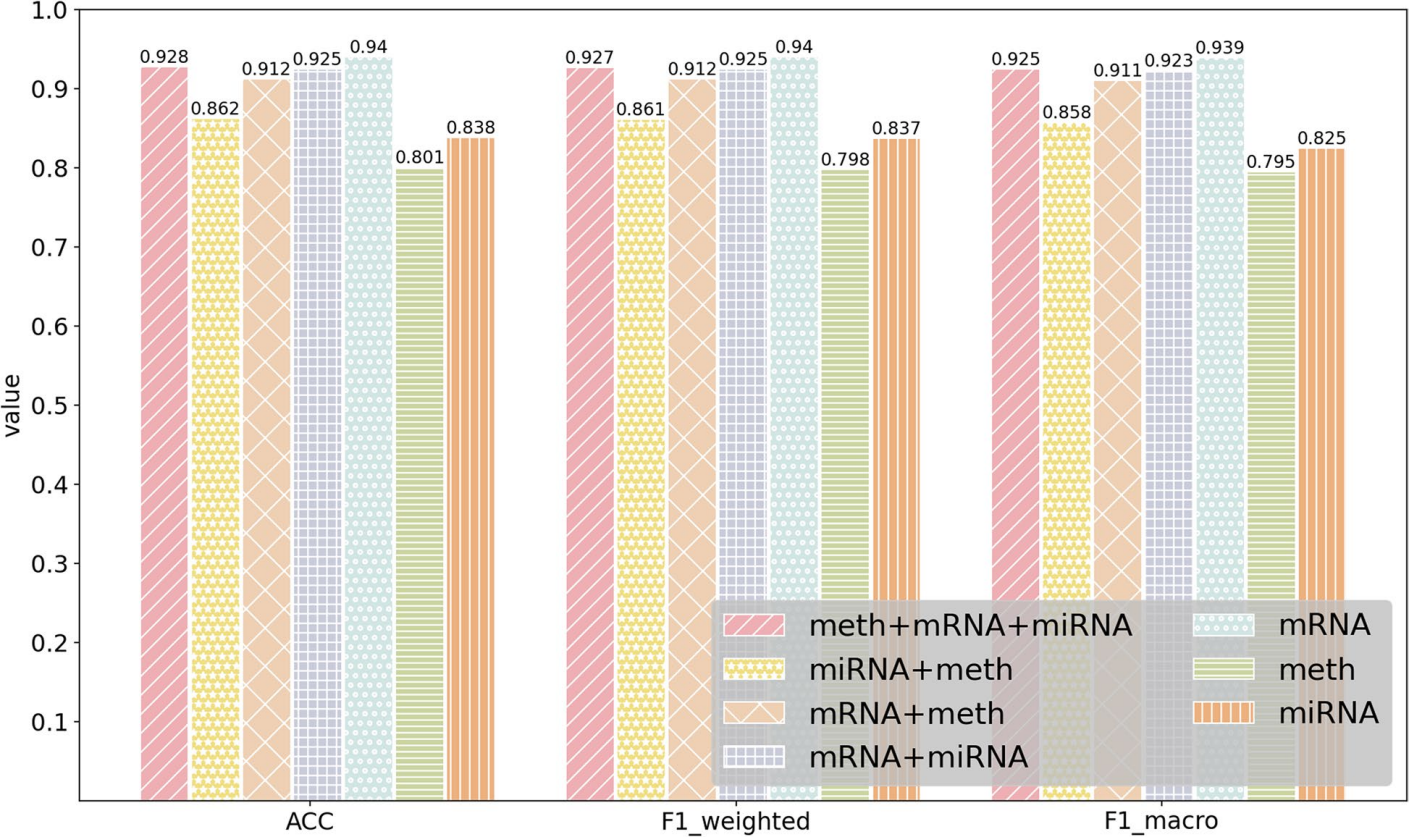
Performance of MODILM with different omics data types in the SKCM dataset

**Fig. 6:**
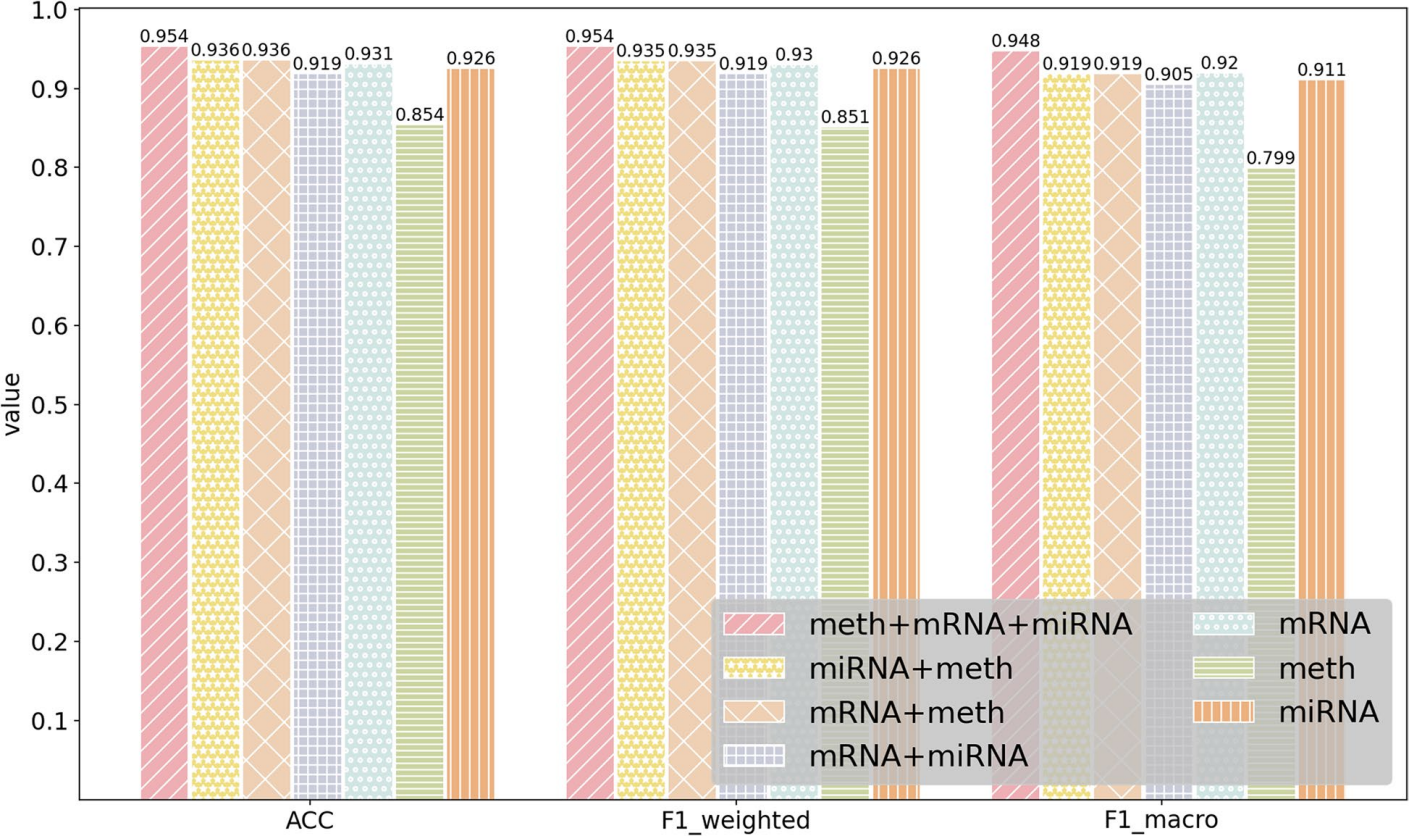
Performance of MODILM with different omics data types in the LGG-4 dataset

**Fig. 7:**
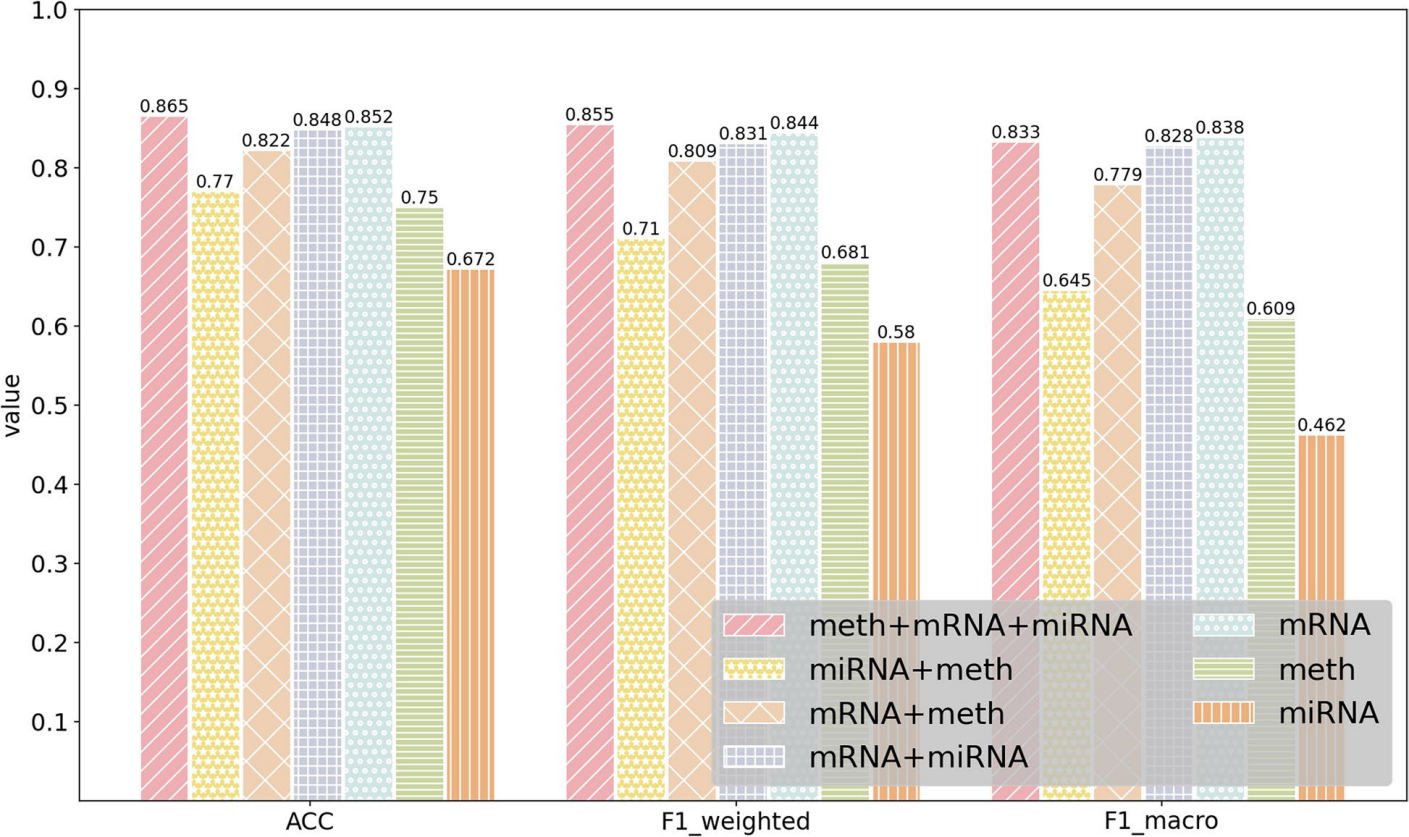
Performance of MODILM with different omics data types in the LUSC dataset

In this section, our experiment investigates which kind of omics data or their combination in MODILM contributes the most to the classification of complex diseases. We build seven types of omics data combinations of comparative experiments using miRNA expression data, mRNA data, and DNA methylation data from each dataset. The experimental results of each dataset under different omics data types are shown in Figs. 2 , 3, 4, 5, 6,  7, indicating that integration of multi-omics data can effectively enhance the classification performance. Specifically, combining three types of omics data yields better classification performance than combining two types of omics data across all datasets. In addition, the results of the combination of various omics types on the LGG-2 dataset are not significantly different. Occasionally, MODILM with specific omics data types (e.g., mRNA in the BRCA and SKCM datasets) can even produce better results than that with the combination of three omics data. There may be two reasons behind this: 1) the contribution of different types of omics data in distinguishing different complex diseases varies; 2) MODILM can capture the important features from all the datasets, thereby improving the performance of classification. This further illustrates the effectiveness of the proposed method.

### The performance influence of some key hyperparameters

1. Influence of the average number of edges retained by each node

When constructing the cosine similarity networks of samples, $k$ (the average number of edges retained by each node) can be used to control the sparsity of the number of edges in the graph, thus allowing it to accurately capture the interactions between samples. This provides extra information on the relevance of the samples and boosts the performance of the model.

**Fig. 8:**
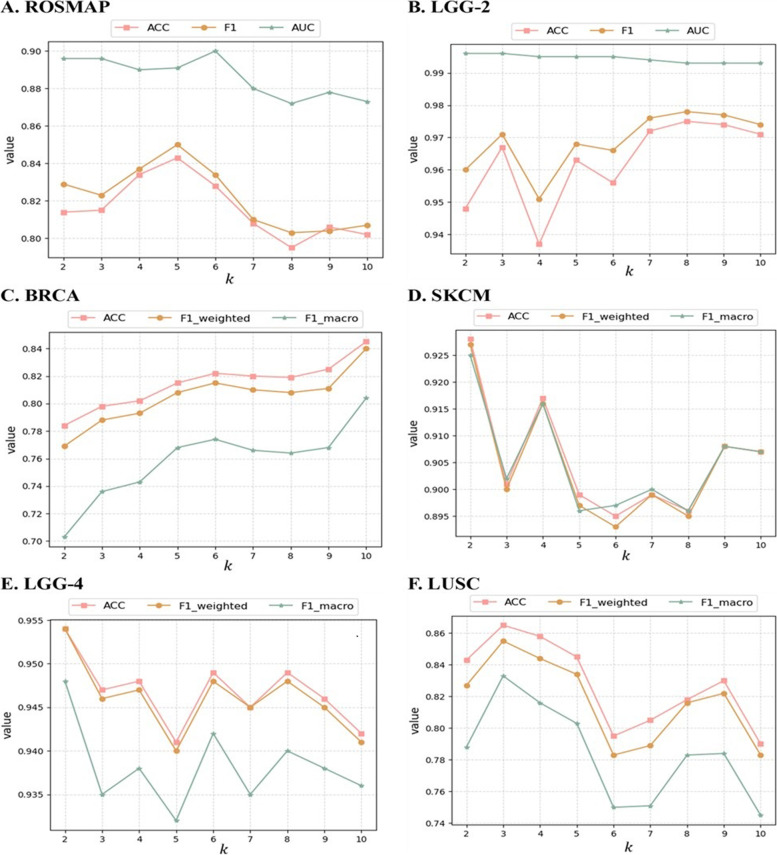
Performance comparison of MODILM with different $k$. **A** Results on the ROSMAP dataset; **B** Results on the LGG-2 dataset; **C** Results on the BRCA dataset; **D** Results on the SKCM dataset. **E** Results on the LGG-4 dataset; **F** Results on the LUSC dataset)

This section focuses on the effects of $k$ on MODILM 's performance. We build MODILM for each dataset with a range of $k$ values to find the optimal one. The model's performance for each dataset when $k$ varies from 2 to 10 is depicted in Fig. 8. The results show that MODILM correspondingly has the best classification result on ROSMAP, LGG-2, BRCA, SKCM, LGG-4 and LUSC datasets when $k$=5,  $k$=8,  $k$=10,  $k$=2,  $k$=2, and $k$=3, repectively. These experimental results demonstrate that the hyperparameter $k$ does affect the classification performance of MODILM and varies with the change of $k$. If $k$ is too low, the similarity network becomes too sparse and some significant interactions between samples may be overlooked. Conversely, if $k$ is too high, the similarity network becomes too dense and the correlations between samples may be contaminated by noise or human factors. The appropriate choice of $k$ depends on the topology of the data, which may vary from dataset to dataset.

Therefore, it is important to select the appropriate $k$ for different datasets.

(2) Influence of the number of GAT layers

In this section, we investigate the influence of the number of GAT layers on MODILM’s performance. We build MODILM models setting the number of GAT layers to 2, 3 and 4 on each dataset, respectively. The results are shown in Table 4.

**Table 4:** Performance investigation of the model with different numbers of GAT layers

Dataset	Method	ACC	F1	AUC	F1_weighted	F1_macro
ROSMAP	**2-layer MODILM (Ours)**	**0.843**	**0.850**	**0.891**	**–-**	**–-**
	3-layer MODILM	0.803	0.811	0.875	**–-**	**–-**
	4-layer MODILM	0.731	0.756	0.839	**–-**	**–-**
LGG-2	**2-layer MODILM (Ours)**	**0.975**	**0.978**	**0.993**	**–-**	**–-**
	3-layer MODILM	0.928	0.946	0.917	**–-**	**–-**
	4-layer MODILM	0.849	0.883	0.919	**–-**	**–-**
BRCA	**2-layer MODILM (Ours)**	**0.845**	**–-**	**–-**	**0.840**	**0.804**
	3-layer MODILM	0.809	**–-**	**–-**	0.795	0.713
	4-layer MODILM	0.765	**–-**	**–-**	0.731	0.612
SKCM	**2-layer MODILM (Ours)**	**0.928**	**–-**	**–-**	**0.927**	**0.925**
	3-layer MODILM	0.902	**–-**	**–-**	0.902	0.903
	4-layer MODILM	0.894	**–-**	**–-**	0.895	0.894
LGG-4	**2-layer MODILM (Ours)**	**0.954**	**–-**	**–-**	**0.954**	**0.948**
	3-layer MODILM	0.881	**–-**	**–-**	0.865	0.838
	4-layer MODILM	0.795	**–-**	**–-**	0.769	0.748
LUSC	**2-layer MODILM (Ours)**	**0.865**	**–-**	**–-**	**0.855**	**0.833**
	3-layer MODILM	0.768	**–-**	**–-**	0.687	0.626
	4-layer MODILM	0.750	**–-**	**–-**	0.677	0.621

Table 4 shows that the performance for each metric in both the binary- and multi-classification tasks is the best when the number of GAT layers is set to 2. As the number of GAT layers gradually increases, the performance of MODILM decreases. It reveals that GAT would be over-smoothing if the number of GAT layers is too large.

Therefore, MODILM can achieve the best performance when the number of GAT layers is set to 2 in this work.

(3) Influence of the number of hidden layers of MLP

**Fig. 9:**
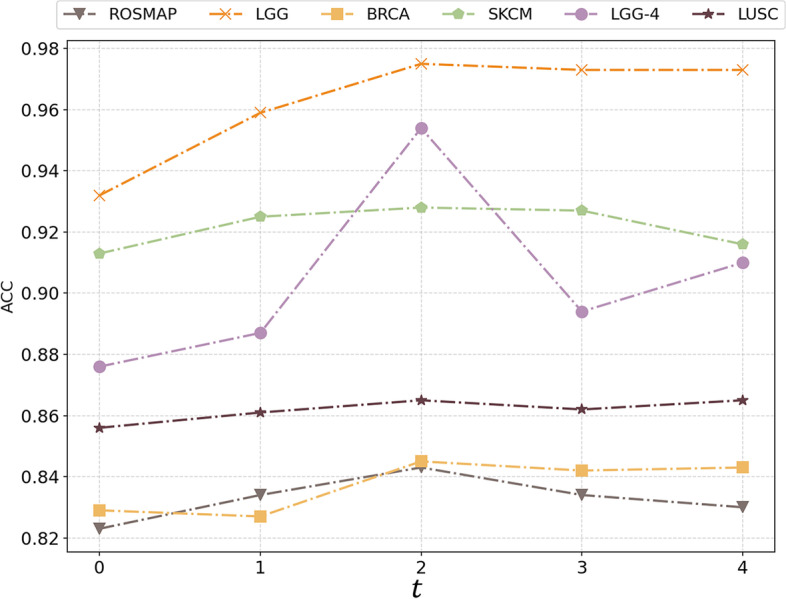
The classification accuracy of MODILM with different $t$ on the benchmark datasets

To investigate the influence of $t$ (the number of hidden layers of the MLP in MODILM) on MODILM performance, we set $t$ from 0 to 4 for each dataset. Note that $t=0$ means that only the features are linearly varying. Figure 9 shows the ACC of MODILM on each dataset. As can be seen in Fig. 9, the ACC of each dataset is best when $t$ is 2. This may be due to the very limited number of omics data samples that can be trained, so the deeper the MLP, the more likely all node features will converge to a fixed point, increasing the risk of overfitting.

Therefore, in this work, we finally set the number of hidden layers to 2.

### Ablation study

1. The performance influence of GAT in MODILM

This section will investigate the impact of the GAT component on the performance of MODILM. To do this, we designed a MODILM–GAT model, which removes the GAT component from MODILM and directly maps the omics features to an MLP model using a simple linear transformation method.

We conducted experiments comparing the MODILM–GAT and MODILM models on each dataset. The experimental results, shown in Table 5, indicate that the classification accuracy of MODILM is much better than that of MODILM–GAT in terms of classification accuracy on all datasets. It suggests that the GAT component plays an important role in MODILM. This is because the GAT component, through its graph convolutional network and attention mechanism, can learn richer single data features, which helps to improve the classification performance of MODILM.

(2) The performance influence of VCDN in MODILM

**Table 5:** The performance influence of GAT in MODILM (ACC)

Setting	ROSMAP	LGG-2	BRCA	SKCM	LGG-4	LUSC
**MODILM**	**0.843**	**0.975**	**0.845**	**0.928**	**0.954**	**0.865**
MODILM–GAT	0.808	0.885	0.780	0.894	0.932	0.780

This section will investigate the impact of the VCDN component on the performance of MODILM. To do this, we designed a MODILM–VCDN model, which removes the VCDN component from MODILM and directly only multiplies multiple omics feature matrices with Hadamard products to integrate multiple omics features.

We conducted experiments comparing the MODILM–VCDN and MODILM models in terms of ACC on each dataset. The experimental results, shown in Table 6, indicate that the classification accuracy of MODILM is much better than that of MODILM–VCDN on all datasets. It reveals that the VCDN component plays an important role in MODILM. This may be because the VCDN component can learn the correlation of the intra- and cross-views at higher levels in the label space.

**Table 6:** The performance influence of VCDN in MODILM (ACC)

Setting	ROSMAP	LGG-2	BRCA	SKCM	LGG-4	LUSC
**MODILM**	**0.843**	**0.975**	**0.845**	**0.928**	**0.954**	**0.865**
MODILM–VCDN	0.565	0.895	0.740	0.785	0.875	0.855

## Conclusion

In this work, we proposed a novel multi-omics data integration learning model, called MODILM, for more accurate classification of complex diseases. MODILM first uses similarity networks and GAT networks to learn important intra-view features for each type of omics data and uses MLP to map these features into a unified feature space for high-level omics-specific feature extraction. MODILM then uses VCDN networks to fuse omics-specific features and learn cross-view correlations in the label space for accurate complex disease classification. Extensive experiments were conducted on six benchmark datasets to evaluate the performance of the proposed model against state-of-the-art models. The results of the experimental comparison show that MODILM achieves state-of-the-art performance across all metrics in all tasks. It indicates that our MODILM can improve complex disease classification tasks by exploring the internal correlations between different omics data, which makes a great contribution to the accurate diagnosis of diseases.

However, the number of layers of GATs in MODILM needs to be set appropriately, otherwise, the model performance may be degraded. This is because increasing the number of layers in the GATs will increase the risk of over-fitting and over-smoothing of MODILM. In the future, we will investigate some new methods to address the problem of model over-smoothing and further improve the performance of complex disease classification tasks.

## Supplementary Information

**Additional file 1.**

## Data Availability

The ROSMAP dataset was obtained from AMP-AD Knowledge Portal (https://adknowledgeportal.synapse.org/). Omics data of LGG-2, LGG-4, BRCA, SKCM and LUSC were obtained from The TCGA public data at Xena website (http://xena.ucsc.edu/). The source code is available from the author on reasonable request.
