# Supplementary material for: MODILM: towards better complex diseases classification using a novel multi-omics data integration learning model
Source: BMC Med Inform Decis Mak. 2023 May 5;23:82. doi: 10.1186/s12911-023-02173-9 (PMC10161645; doi:10.1186/s12911-023-02173-9)
Supplement: Supplementary file 1 — Additional file 1. [file 12911_2023_2173_MOESM1_ESM.pdf]

MODILM: Towards Better Complex Diseases Classification Using a Novel Multi-omics Data Integration Learning Model

Yating Zhong[1], Yuzhong Peng[1,*], Yanmei Lin[2,*], Dingjia Chen[1], Hao Zhang[3,4], Wen Zheng[1], Yuanyuan Chen[1], Changliang Wu[5]
Correspondence:jedison@163.com
1 Guangxi Key Lab of Human-machine Interaction and Intelligent Decision, Nanning Normal University, Nanning 530001, China
2 School of environment and life science, Nanning Normal University, Nanning 530001, China
3 School of Computer Science, Fudan University, Shanghai 200433, China
4 School of Computer, Guangdong University of Petrochemical Technology, Maoming 525000, China
5 Department of Spleen, Stomach and Liver diseases, Guangxi International Zhuang Medical Hospital, Nanning 530201, China

The hyperparameters settings of P-NET, MOMA, and MOGONET:

(1) P-NET: P-NET is a self-defined feedforward neural network model. We set the hidden_layers of the model to 5 and the dropout rate for each hidden layer is set to [0.5, 0.1, 0.1, 0.1, 0.1]. We used Adam as the optimization function, and the activation function is Tanh. The learning rate is set to 0.001, the epoch is set to 500, and batch_size is set to 100.

(2) MOMA: module encoder is set for the MOMA model. For these models, each dataset has a different number of modules and a different learning rate. On the ROSMAP dataset, the number of modules is set to 64 and the learning rate is set to 5e-6. The number of modules in the LGG-2 dataset is set to 32, and the learning rate is set to 5e-5. The number of modules in the BRCA dataset is set to 64, and the learning rate is set to 5e-7. The number of modules in the SKCM dataset is set to 128, and the learning rate is set to 5e-5. The number of modules in the LGG-4 dataset is set to 64, and the learning rate is set to 5e-5. The number of modules on the LUSC dataset is set to 64 and the learning rate is set to 5e-7. We used Adam as the optimization function, and the activation function is Relu. The weight decay is set to 1e-4, and the epoch is set to 5000.

(3) MOGONET: MOGONET mainly uses GCN as the backbone network. We set the number of layers of GCN to 3, and use Adam as the optimization function, the activation function Leaky_Relu, the learning rate to 5e-5, and the dropout rate to 0.5, the epoch is set to 3000.
